# Enhancing Fuel Cell Performance by Constructing a Gas Diffusion Layer with Gradient Microstructure

**DOI:** 10.3390/ma18143271

**Published:** 2025-07-11

**Authors:** Rui-Xin Wang, Bai-He Chen, Ye-Fan-Hao Wang, Cheng Guo, Bo-Wen Deng, Zhou-Long Song, Yi You, Hai-Bo Jiang

**Affiliations:** Shanghai Engineering Research Center of Hierarchical Nanomaterials, Key Laboratory for Ultrafine Materials of Ministry of Education, School of Materials Science and Engineering, East China University of Science and Technology, Shanghai 200237, China; 17662626166@163.com (R.-X.W.);

**Keywords:** proton exchange membrane fuel cell (PEMFC), gas–liquid transmission, gas diffusion layer (GDL), multi-scale pore structure

## Abstract

This study focuses on addressing the issues of water flooding and mass transfer limitations in proton exchange membrane fuel cells (PEMFCs) under high current density conditions. A multi-scale gradient pore gas diffusion layer (GDL) is designed to enhance fuel cell performance. The pore structure is precisely controlled using a self-assembled mold, resulting in the fabrication of a GDL with a gradient distribution of pore diameters ranging from 80 to 170 μm. Experimental results indicate that, with the optimized gradient pore GDL, the peak power density of the fuel cell reaches 1.18 W·cm−^2^, representing a 20% improvement compared to the traditional structure. A mechanism analysis reveals that this structure establishes a concentrated water transport pathway through channels while enabling gas diffusion and transport driven by concentration gradients, thereby achieving the collaborative optimization of gas–liquid transport. This approach offers a novel solution for managing water in PEMFCs operating under high current density conditions, and holds significant implications for advancing the commercialization of PEMFC technology.

## 1. Introduction

With the ongoing deterioration of the global environmental, pollution, and the accelerating depletion of fossil fuel resources, the development of electrochemical energy conversion technologies that are both sustainable and renewable has emerged as a cutting-edge research focus in the international scientific community [1]. Proton exchange membrane fuel cells (PEMFCs) exhibit advantages, such as high energy conversion efficiency, high energy density, and clean, pollution-free operation, which are of significant importance for the development of future societal renewable energy systems. Among these components, the gas diffusion layer (GDL) plays a crucial role in determining the overall performance and efficiency of the fuel cell. It not only provides essential pathways for reactant gases [2] but serves as a key channel for electron transport and water management. Furthermore, the GDL supports the catalytic layer and contributes to maintaining the structural stability of the electrode [3].

During the operation of a proton exchange membrane fuel cell (PEMFC), performance degradation resulting from phenomena such as activation overpotential, ohmic overpotential, and concentration overpotential is inevitable [4]. The typical polarization f binder and compression on the curve of a PEMFC indicates that the open-circuit voltage is lower than the theoretical value [5], primarily due to gas crossover and electrochemical polarization losses [6]. At high current densities, transport phenomena become critical. During fuel cell operation, the reverse transmission of the gas–liquid two-phase flow can lead to restricted gas supply and inadequate water removal, thereby causing “flooding” issues and degrading cell performance [3].

The GDL serves as a critical component for water management in fuel cells [7], and its structural design significantly influences the efficiency of water and gas transport within the diffusion layer, thereby impacting the mass transfer efficiency of the fuel cells. Kong et al. [8] investigated the influence of pore size distribution on gas transport and fuel cell performance, concluding that pore size distribution, as a critical structural parameter, significantly affects fuel cell performance. Specifically, water removal occurs through larger pores, while gas diffusion is facilitated via smaller pores. By comparing an unsaturated flow theory (UFT) model with a two-phase flow model (M^2^),Pasaogullari and Wang et al. [9] investigated the impact of gas phase pressure distribution on two-phase transport within the GDL. They further proposed that liquid saturation in the catalyst layer could be reduced by altering the thickness, porosity, and wettability characteristics of the microporous layer (MPL). WANG et al. [10] systematically investigated the two-phase behavior of the GDL under compression with varying porosity using the two-dimensional lattice Boltzmann method. They discovered that adjusting the porosity gradient could effectively reduce the liquid water saturation within the GDL.

The conventional GDL preparation method is relatively limited, making it challenging for batteries to maintain stable performance across varying operating conditions. Recently, numerous scholars have investigated diverse GDL modification approaches. He et al. [11] developed an integrated GDL via laser carving, incorporating waveform flow channels and micro-channel ridges. This design achieved 80% of pure oxygen performance at 0 kPa back pressure, with power density stabilizing at an optimal value over a broad humidity range (40–100% RH). Nevertheless, the structure of this novel GDL is relatively intricate, requiring the precise control of multiple parameters during fabrication. This complexity may compromise production consistency and stability. Additionally, its durability and performance degradation under prolonged operation remain to be further validated. Ren et al. [12] developed nano-graded diffusion layers (nano-GDLs) featuring uniform pore sizes and three-, four-, and five-layer gradient pore structures. The gradient pore architecture was fabricated using electrospinning technology. It was determined that the breakthrough pressure and water saturation of the three-layer gradient structure (3-MG) were optimal, leading to a 14% increase in power density without cathode humidification. Nevertheless, under high humidity conditions, the advantages of the gradient pore nano-GDL are diminished, and excessive water retention may result in liquid water accumulation, thereby impacting the performance of fuel cells. Zhang et al. [13] investigated the dual hydrophobic CCL structure characterized by an inner hydrophilic layer and an outer hydrophobic layer. By incorporating an appropriate amount of NH_4_HCO_3_ as a pore-forming agent, they demonstrated that simultaneously optimizing the porosity and hydrophobicity of the cathode catalyst layer could substantially enhance the performance of PEMFCs. Nevertheless, the use of the pore-forming agent [14] might potentially compromise the compatibility of the catalyst layer with other components, such as proton exchange membranes, as well as the overall stability of the system. Zahiri et al. [15] developed a wettability gradient on a carbon fiber substrate through a single-step oxygen plasma treatment [16] and evaluated the wettability of the sample via static contact angle measurements and electrochemical double-layer capacitance analysis. While plasma treatment can penetrate into the interior of porous carbon materials, its depth is typically restricted, with wettability alterations primarily occurring at the material’s surface. Additionally, the complexity of the plasma treatment process poses challenges for its application in large-scale production scenarios.

**Figure 1:**
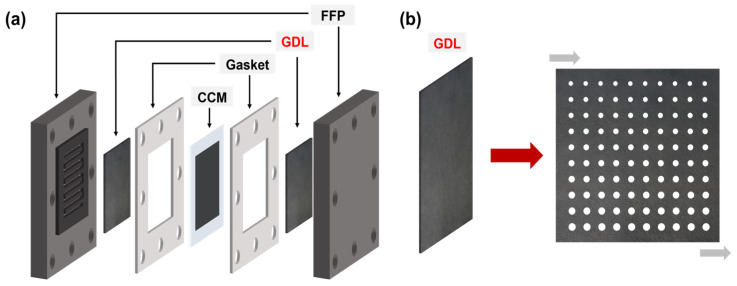
(**a**) Schematic illustration of the proton exchange membrane fuel cell architecture. (**b**) Diagram of the gas diffusion layer pore structure.

In practical applications, the modification of the GDL must be both simple and efficient to reduce manufacturing costs and operating time [15]. Consequently, this study proposes a rapid method for preparing a high-performance porous gas diffusion layer. Given that water flooding typically occurs on the cathode side, the cathode gas diffusion layer was selected for perforation treatment (the schematic diagram of GDL modification is shown in Figure 1). Furthermore, a self-assembly mold was employed to control the pore size and pore spacing, thereby enabling precise regulation of the gas diffusion layer’s pore structure. By comparing the performance curves of the gas diffusion layers with varying pore structures and calculating the effective oxygen diffusion coefficient, it was demonstrated that the gradient pore structure generally outperforms the uniform pore structure. Specifically, a larger pore size distribution near the gas inlet side is more advantageous for enhancing fuel cell performance. Manke et al. [17] carried out an in situ quantitative investigation into the local saturation of operating batteries by means of neutron imaging. The study demonstrated that local saturation has a decisive impact on oxygen transport resistance and confirmed the efficacy of perforation in reducing saturation through enhanced drainage. The pore structure design proposed in this study effectively addresses water management challenges under high current density conditions and significantly improves the performance stability of fuel cells [18].

## 2. Materials and Methods

### 2.1. Hydrophobic Modification of the Carbon Paper

The polytetrafluoroethylene (PTFE) emulsion (60 wt%) [DAIKIN INDUSTRIES, LTD , Osaka, Japan] was diluted to 10 wt% with deionized water and subsequently dispersed via ultrasonication for 10 min [19]. The Toray carbon paper (YLS-30T, 190 μm thickness) [Suzhou Sinero Technology Co., Ltd., Suzhou, China] was cut into 2 cm × 2 cm pieces. A 20 mL acetone solution (analytical grade) was prepared, and the carbon paper was ultrasonicated in this solution for 20 min. Afterward, the carbon paper was retrieved, placed on a clean glass dish, and dried in an oven at 60 °C for 30 min. Subsequently, the carbon paper was ultrasonicated in 20 mL of ethanol [Shanghai Macklin Biochemical Technology Co., Ltd., Shanghai, China] for 20 min and then dried again in the oven at 60 °C. The carbon paper was subsequently immersed repeatedly in the diluted PTFE emulsion until the mass of PTFE accounted for 30% of the carbon paper’s total mass. Finally, the treated carbon paper was placed in a Muffle furnace [20], where it underwent heat treatment at a heating rate of 10 °C/min and was maintained at 350 °C for 60 min.

### 2.2. Preparation of the Microporous Layers

Carbon black (98 mg, Vulcan XC-72R), PTFE suspension (420 mg, 10 wt%), deionized water (9.8 g), and isopropyl alcohol (19.60 g, analytical grade) were subjected to ultrasonic treatment for 2 h to form a homogeneous slurry [21]. This slurry was subsequently sprayed uniformly onto the surface of the hydrophobic carbon paper via ultrasonic atomization. The resulting coating exhibited a loading of 2 mg/cm^2^. Finally, the microporous layer was fabricated by subjecting the coated carbon paper to heat treatment in a muffle furnace (Hefei Kejing Materials Technology Co., Ltd., Hefei, China) at 350 °C for 60 min [19]. The commercial carbon paper used in this research conforms to the standard of the MPL-coated GDL, with no gap existing between the two components.

### 2.3. Construction of the Multi-Scale Pore Structures

**Figure 2:**
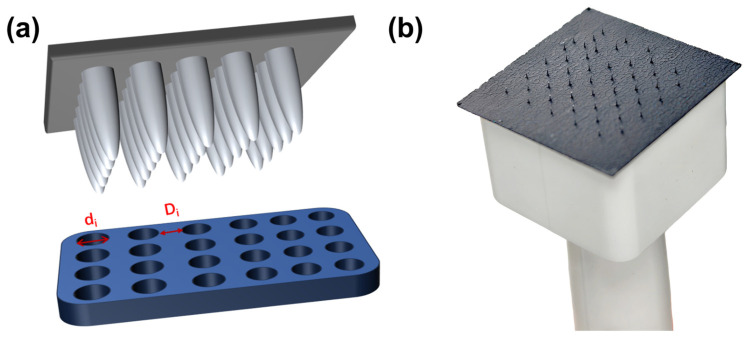
(**a**) Puncture diagram; (**b**) mold structure diagram.

**Figure 3:**
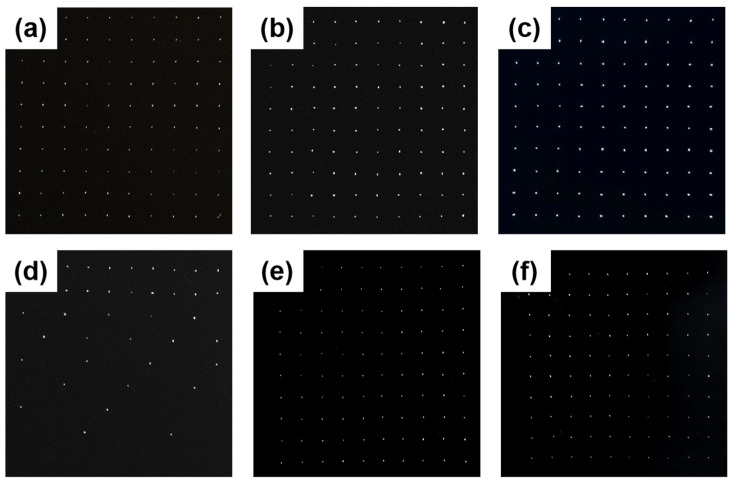
Structure diagram of the gas diffusion layer: (**a**) carbon paper with a uniformly distributed aperture of approximately 30 μm in diameter; (**b**) carbon paper featuring a uniform pore diameter of approximately 70 μm; (**c**) carbon paper with a consistent aperture size of approximately 120 μm in diameter; (**d**) carbon paper with a consistent aperture size of approximately 80 μm in diameter and a spacing gradient ranging from 1750 to 7000 μm; (**e**) carbon paper with a diameter gradient ranging from approximately 50 to 140 μm and a fixed spacing of 1750 μm; (**f**) carbon paper with a diameter gradient decreasing from approximately 140 to 50 μm and a fixed spacing of 1750 μm.

Figure 2a presents the puncture mold diagram, while Figure 2b illustrates the mold structure. Figure 3 depicts the gas diffusion layer with multi-scale pore distribution prepared by controlling the needle diameter and inter-needle distance of the puncture mold. To fabricate a gas diffusion layer with uniform pore distribution, the carbon paper was placed on a silicone rubber plate. The steel needle spacing was fixed at 1750 μm, and the diameter of the mold steel needles was varied. Using a press, the mold was punctured under 30 kg pressure, resulting in structures with uniform aperture distributions of approximately 30 μm, 70 μm, and 120 μm, as shown in Figure 3a–c. For preparing a gas diffusion layer with a gradient of pore size and equal spacing, steel needles with a fixed diameter of 80 μm were employed in the experiment. The spacing between the adjacent rows of steel needles was systematically varied to 1750 μm between the first and second rows, 3500 μm between the third and fourth rows, 5250 μm between the fifth and sixth rows, and 7000 μm between the seventh and eighth rows. The ninth and tenth rows remained unprocessed. The positioning of the steel needles on the mold was relatively fixed due to the design constraints of the mold, which limited the spacing adjustments to incremental multiples. Under 30 kg pressure, carbon paper was punctured to achieve a structure with a gradient of pore spacing, as depicted in Figure 3d. To obtain a gas diffusion layer with a gradient of pore size, the diameter of the die steel needle was gradually increased from 50 μm to 140 μm (with each increment being 10 μm), while the inter-needle distance remained constant at 1750 μm. Carbon paper was positioned on the silicone rubber plate, and a press set to 30 kg was used for puncturing, yielding the structure shown in Figure 3e. Finally, for achieving another gas diffusion layer with a gradient of pore size, the diameter of the die steel needle was progressively reduced from 140 μm to 50 μm (decreasing by 10 μm each time), with the inter-needle distance remaining unchanged at 1750 μm. Carbon paper was placed on the silicone rubber plate, and a press set to 30 kg was utilized for puncturing, resulting in the structure illustrated in Figure 3f.

### 2.4. Single Cell Assembly and Performance Testing

The cathode diffusion layer features either a uniform pore structure or a gradient pore structure, while the anode diffusion layer adopts a conventional structure. The catalyst-coated membrane (CCM) (W. L. Gore & Associates, Newark, DE, USA) utilized in the experiment was from the Gore PRIMEA series [22]. The platinum loading on the anode and cathode was 0.05 mg/cm^2^ and 0.4 mg/cm^2^, respectively. These components were assembled into a single cell for testing. The anode hydrogen flow rate was set to 110 mL/min, while the cathode air flow rate was adjusted to 419 mL/min. Both gas streams passed through a humidifier maintained at 70 °C. The operating temperature of the fuel cell [23] was also set to 70 °C. Back pressure was adjusted according to the experimental requirements, and a Chroma 6312A (Taiwan Zhimo Electronics Co., Ltd., Taiwan, China) electronic load was employed to record the voltage under various current densities.

## 3. Results and Discussions

### 3.1. Morphological Characteristics and Structural Composition of the Diffusion Layer

**Figure 4:**
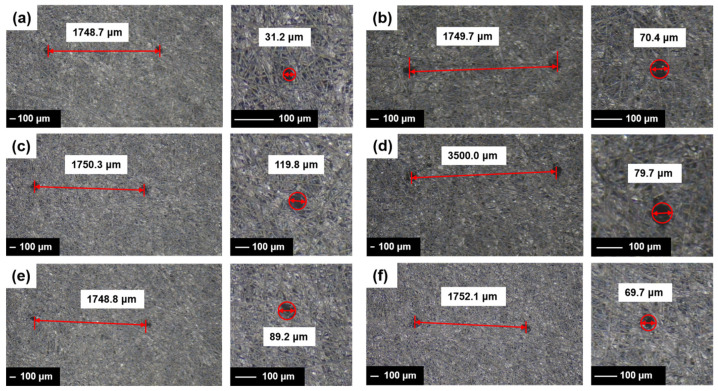
Digital photographs of the GDL with varying needle diameters and spacing configurations: (**a**) 30 μm needle diameter with 1750 μm spacing; (**b**) 70 μm needle diameter with 1750 μm spacing; (**c**) 120 μm needle diameter with 1750 μm spacing; (**d**) 80 μm needle diameter with variable spacing ranging from 1750 μm to 7000 μm; (**e**) needle diameter ranging from 50 μm to 140 μm with 1750 μm spacing; (**f**) needle diameter decreasing from 140 μm to 50 μm with 1750 μm spacing.

The GDL featuring a multi-scale structure was fabricated by adjusting the diameter and spacing of the steel needles. The pore distribution of the GDL was imaged using a digital camera (Shenzhen Shua’an Technology Development Co., Ltd., Shenzhen, China), and the aperture size and spacing were quantitatively analyzed using e-ruler software. Figure 4 presents the data regarding the size and spacing of the selected pores (corresponding to the diffusion layer depicted in Figure 3).

### 3.2. Single-Cell Performance Analysis

**Figure 5:**
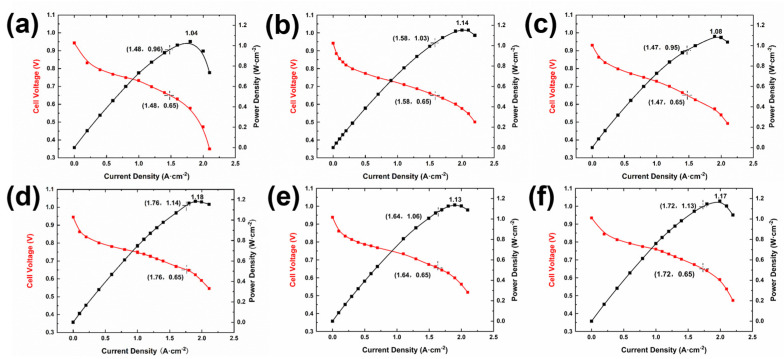
The polarization curves and power density curves of the gas diffusion layer (GDL) with varying pore structures are presented as follows: (**a**) GDL structure featuring cells with a 30 μm pin diameter and 1750 μm spacing; (**b**) GDL structure consisting of cells with a 70 μm needle diameter and 1750 μm spacing; (**c**) GDL structure composed of cells with a 120 μm needle diameter and 1750 μm spacing; (**d**) GDL structure including cells with an 80 μm needle diameter and variable spacing ranging from 1750 μm to 7000 μm; (**e**) GDL structure comprising cells with needle diameters ranging from 50 μm to 140 μm and fixed 1750 μm spacing; (**f**) GDL structure involving cells with needle diameters decreasing from 140 μm to 50 μm and fixed 1750 μm spacing.

The GDL with varying structures were integrated into single cells using Gore’s CCM (W. L. Gore & Associates, Newark, DE, USA) and bipolar plates, and the performance of these single cells was subsequently evaluated. The test results are presented in Figure 5. As shown in Figure 3, the fuel cells assembled with the GDLs featuring pore structures exhibited superior performance compared to those without special treatment. Notably, the single cell utilizing the GDL structure corresponding to sample d in Figure 3 demonstrated the highest performance, achieving a peak power density of 1.18 W·cm−^2^, approximately 20% higher than that of the untreated sample. By comparing Figure 5a–c, it is evident that the single cell with the GDL structure of sample b performed best, reaching a peak power density of 1.14 W·cm−^2^. This suggests that, for GDLs with uniformly distributed pores, both excessively large and small pore diameters are detrimental to enhancing fuel cell performance. Based on the single-cell performance test results, it can be inferred that an appropriate pore size is essential for improving fuel cell performance. Furthermore, the overall performance of the GDLs with gradient pore distribution (Figure 3d–f) surpasses that of the samples with uniform pore distribution (Figure 3a–c). This is primarily attributed to the MPL with a gradient pore structure, which effectively reduces the contact resistance between the catalyst layer (CL) and the GDL [24]. By comparing Figure 5e,f, it is clear that, under identical hole spacing conditions, placing the side with the larger hole diameter at the air inlet yields better performance than positioning the side with the smaller hole diameter at the air inlet. Consequently, the peak power density of the fuel cell increases from 1.13 W·cm−^2^ to 1.17 W·cm−^2^, representing a 3.54% improvement.

Based on the polarization curve presented in Figure 5, the limiting current density *i_L_* was determined. By combining this value with the calculation formula for the effective diffusion coefficient of oxygen, the effective oxygen diffusion coefficient of the gas diffusion layer can be calculated by the following equation:

(1) $$i_{L}=nFD^{eff}\frac{c_{R}^{0}}{\delta }$$

**Figure 6:**
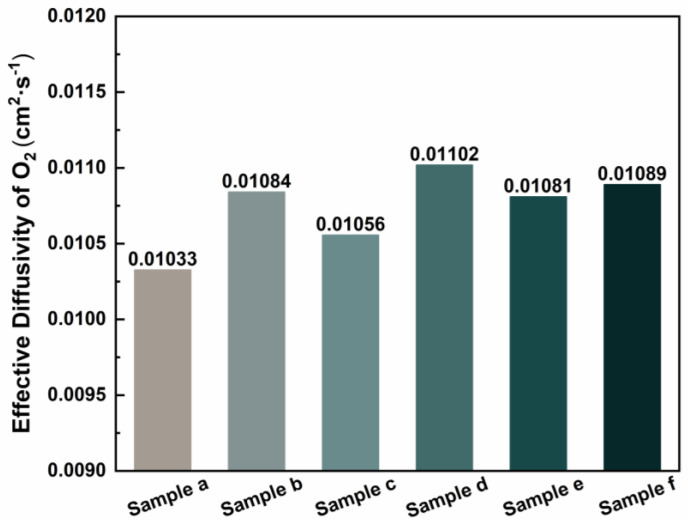
Effective oxygen diffusion coefficient.

The value for *n* = 4, the Faraday constant, and the diffusion layer thickness delta = 190 μm = 0.019 cm, the effective diffusion coefficient *D_eff_* of oxygen was calculated. The results for each sample are illustrated in Figure 6. The conclusions drawn from the polarization curve (Figure 5) were further validated by calculating the effective diffusion coefficients of oxygen for the different samples. Notably, the effective diffusion coefficient of oxygen in the sample depicted in Figure 5d is the highest, reaching 0.01102 cm^2^ s^−1^. Compared to samples a–c, samples d–f exhibit higher overall effective diffusion coefficients of oxygen, indicating reduced migration resistance of oxygen molecules within the material. This allows for faster diffusion of oxygen from the flow channel to the catalytic layer, thereby reducing the concentration gradient and significantly decreasing concentration polarization. This characteristic directly enhances the efficiency of the oxygen supply and mitigates the local hypoxia caused by mass transfer limitations. During the operation of a fuel cell, electrochemical reactions initially occur at the gas inlet, resulting in the rapid generation of a significant amount of water [25] that accumulates on one side of the gas inlet [26]. The process of water intrusion is governed by the capillary fingering mechanism, and the distribution and dynamic behavior of water are significantly influenced by pore-scale wettability and porosity distribution [27]. Sample e features a pore size gradient distribution structure; however, the smaller pore sizes are positioned on the air inlet side. Water accumulation may obstruct the oxygen transport pathway, resulting in localized oxygen concentration elevation and influencing the electrochemical reaction process [27], which hinders the efficient discharge of water molecules. Consequently, its ability to enhance fuel cell performance is restricted. By contrast, the larger pore size distribution structure of sample f effectively facilitates the rapid discharge of water generated at the cathode gas inlet [28], significantly mitigating the issue of water flooding in the fuel cells. The microfluidic visualization study [29] demonstrated the physical regulation of perforations on the capillary fingering pathway. Their experimental video evidence revealed that the droplets exhibited an organized ejection pattern at the channel outlets—an observation derived directly from the empirical data rather than theoretical modeling—thereby aligning precisely with the mechanism of directional droplet migration identified in this study.

Therefore, a pore-making treatment applied to the GDL can promote water discharge via the pores while ensuring adequate gas diffusion through the carbon fiber membrane, thereby achieving the relative separation of water vapor transmission.

## 4. Conclusions

This study successfully developed a GDL with a multi-scale gradient pore structure, demonstrating significant performance enhancements in PEMFCs. The key findings are as follows:

(1) the optimized gradient pore design (80→170 μm, with larger pores near the gas inlet) achieved a peak power density of 1.18 W·cm−^2^, representing a 20% improvement over conventional GDLs;
(2) the structure effectively decoupled gas and liquid transport, reducing oxygen diffusion resistance (effective diffusion coefficient up to 0.01102 cm^2^·s−^1^) and mitigating flooding issues;
(3) a scalable self-assembly mold method was established for precise pore structure control.

The proposed GDL design addresses critical mass transfer limitations in PEMFCs, offering practical solutions for high current-density applications in transportation and stationary power systems. Future work should focus on dynamic performance validation, systematic parameter optimization (e.g., gradient slope), and integration of advanced nanomaterials to further enhance durability and efficiency.

While this study provides fundamental insights into structure–performance relationships, limitations include the lack of long-term stability tests under variable loads and a comprehensive cost analysis for industrial scaling. These aspects warrant further investigation to facilitate commercial adoption. The findings advance porous electrode engineering and contribute to the development of next-generation fuel cell technologies.

## Data Availability

The original contributions presented in this study are included in the article. Further inquiries can be directed to the corresponding author.
